# Supplementary material for: Systematic review of the characteristics of brief team interventions to clarify roles and improve functioning in healthcare teams
Source: PLoS One. 2020 Jun 10;15(6):e0234416. doi: 10.1371/journal.pone.0234416 (PMC7286504; doi:10.1371/journal.pone.0234416)
Supplement: S1 File — (DOCX) [file pone.0234416.s002.docx]

**Appendix. Pubmed search strategy**

("Advanced Practice Nursing"[MH] OR

"nursing, team"[MH] OR

"health personnel"[MH] OR

"patient care team"[MH] OR

"nurse practitioners"[MH] OR

"medical staff, hospital"[MH] OR

"general practice"[MH] OR

teamwork[tiab])

AND

("trust"[MH] OR

"team effectiveness"[tiab] OR

"communication"[MH] OR

"decision making"[MH] OR

"cohesion"[tiab] OR

"problem solving"[MH] OR

"patient-centered care"[MH])

AND

("patient simulation"[MH] OR

"cooperative behavior"[MH] OR

"practice guidelines as topic"[MH] OR

"task performance and analysis"[MH] OR

"proxy"[MH] OR

"education, medical"[MH] OR

"Education, Nursing, Continuing"[MH] OR

"interprofessional relations"[MH] OR

"outcome assessment (health care)"[MH] OR

"delivery of health care"[MH] OR

"primary care nursing"[MH] OR

"inservice training"[MH] OR

"communication"[MH] OR

"interprofessional relations"[MH] OR

"teaching"[MH])

AND

("role clarification"[tiab] OR

"role clarity"[tiab] OR

"role development"[tiab] OR

"professional role"[MH] OR

"nurse's Role"[MH] OR

"models, organizational"[MH] OR

"models, theoretical"[MH])

AND

("randomized controlled trial"[pt] OR

"controlled clinical trial"[pt] OR

"clinical trial"[pt] OR

"meta-analysis"[pt] OR

"systematic review"[tiab] OR

"comparative study"[pt] OR

"drug therapy"[SH])

OR

("Advanced Practice Nursing"[kw] OR

"nursing, team"[kw] OR

"health personnel"[kw] OR

"patient care team"[kw] OR

"nurse practitioners"[kw] OR

"medical staff, hospital"[kw] OR

"general practice"[kw] OR

"teamwork"[kw])

AND

("trust"[kw] OR

"team effectiveness"[kw] OR

"communication"[kw] OR

"decision making"[kw] OR

"cohesion"[kw] OR

"problem solving"[kw] OR

"patient-centered care"[kw] OR

"boundary work"[kw] OR

"team effectiveness"[kw] OR

"efficacy"[kw] OR

"coordination"[kw])

AND

("patient simulation"[kw] OR

"cooperative behavior"[kw] OR

"practice guidelines as topic"[kw] OR

"task performance and analysis"[kw] OR

"proxy"[kw] OR

"education, medical"[kw] OR

"Education, Nursing, Continuing"[kw] OR

"interprofessional relations"[kw] OR

"outcome assessment (health care)"[kw] OR

"delivery of health care"[kw] OR

"primary care nursing"[kw] OR

"inservice training"[kw] OR

"communication"[kw] OR

"interprofessional relations"[kw] OR

"teaching"[kw] OR

"intervention"[kw])

AND

("role clarification"[kw] OR

"role clarity"[kw] OR

"role development"[kw] OR

"professional role"[kw] OR

"nurse's Role"[kw] OR

"models, organizational"[kw] OR

"models, theoretical"[kw])

AND

("randomized controlled trial"[kw] OR

"controlled clinical trial"[kw] OR

"clinical trial"[kw] OR

"meta-analysis"[kw] OR

"systematic review"[kw] OR

"comparative study"[kw] OR

"drug therapy"[kw])

OR

("TEAMWORK TRAINING"[TIAB])
